# Combination of Screen-Printed Carbon Electrode and Molecularly Imprinted Polymers for the Selective Determination of Phenolic Compounds in Wine

**DOI:** 10.3390/antiox11102036

**Published:** 2022-10-15

**Authors:** Marie Elhachem, Elias Bou-Maroun, Maher Abboud, Richard G. Maroun, Philippe Cayot

**Affiliations:** 1 PAM UMR A 02.102, Institut Agro, Université Bourgogne Franche-Comté, 1 Esplanade Erasme, F-21000 Dijon, France; 2 Centre d’Analyses et de Recherche, Laboratoire CTA, UR TVA, Faculty of Sciences, Saint Joseph University, Beirut 1104 2020, Lebanon; 3 UEGP Unité Environnement, Génomique et Protéomique, Faculty of Sciences, Saint Joseph University, BP 17-5208 Mar Mikhael, Beirut 1104 2020, Lebanon

**Keywords:** electrochemistry, molecularly imprinted polymer, screen-printed carbon electrode, caffeic acid, wine, cyclic voltammetry

## Abstract

Caffeic acid (CA) is an efficient antioxidant found in wine and in plants and can be extracted from the by-products of the food industry. A molecularly imprinted polymer specific to caffeic acid (CA-MIP) was prepared by radical polymerization using *N*-phenylacrylamide as the functional monomer, ethylene glycol dimethacrylate as the cross-linker, and azobisisobutyronitrile as the initiator, in the presence of CA as the template molecule. The rebinding activities between the polymers and CA were promoted by an indirect method and characterized by cyclic voltammetry (CV) using a screen-printed carbon electrode (SPCE). It is a fast method, which only requires simple and portable instrumentation. The polymer showed a high selectivity toward CA and a good repeatability. CA-MIP was then applied in wine samples spiked with CA, and the results were compared to those obtained by a chromatographic method. With a limit of detection of 0.06 mM in wine, the recovery values confirmed that the method is suitable for further applications.

## 1. Introduction

Antioxidants (AOs) are of great importance in our lives, whether it is in biology (oxidative stress), medicine (damage by oxidation of RNA and DNA, Alzheimer’s disease, and cancer) [1,2], nutrition (loss of essential fatty acids, toxicological impact of lipid oxidation products, loss of amino acids such as Tryptophan) [3,4], and so, in our personal health. Their main targets are reactive oxygen species (ROSs), such as free radicals (superoxide ion O_2_^•^, peroxide ROO^•^, hydroxyl radical HO^•^, singlet dioxygen ^1^O_2_ or ^•^O O^•^), mainly derived from oxidation reactions. The ROSs target different structures (lipids and particularly unsaturated fatty acid residues, proteins with sensible side chains such as Met, Cys, Lys, Trp, Tyr, Phe, and carbohydrates), which can affect foods and health [5]. Antioxidants (AO H) constitute a family of molecules that react preferentially and readily with radicals. They exchange a single electron and a hydrogen atom with the radical (R^•^): R^•^ + AO H → R H + AO^•^. The radical antioxidant (AO^•^) is stabilized by resonance (dissemination of the free electron on the structure). Sometimes, it polymerizes (AO^•^ + AO^•^ → AO-AO) or gives a non-radical product when it is a diradical (^•^AO^•^ → AO’). Therefore, AOs protect the oxidizable species commonly found in pharmaceuticals, paramedical products, cosmetics, and foods [6]. Particular consideration is given to natural antioxidants, which can be easily found in our nutrition. Caffeic acid, for example, is a very common polyphenol, known for its antioxidant, anti-inflammatory, anti-AIDS, and anticarcinogenic activity. It belongs to the class of phenolic acids, specifically hydroxycinnamic acids. It is widespread in plants and foods such as rosemary, fruits such wild blueberries, blackcurrants, artichokes, black beans, but also coproducts of already-prepared plants and fruits such as potato peels or fruits peel, which can be valorized by antioxidant extraction, and drinks such as ginkgo infusion, tea, wine, etc. [7,8].

The importance of these natural antioxidants makes their determination essential, especially in food products, in order to protect them from oxidation and to provide the consumer with a safe product and good organoleptic qualities. Several methods have been developed for assessing the total antioxidant capacity. Direct methods to measure the antioxidant capacity (AOC, named sometimes antioxidant ability (AOA) or efficiency (AOE)) consist of a continuous record of the oxidation degree of a system during storage. They are based on a comparison between a control without AO and a system containing AO, under normal or accelerated conditions (for example, the RANCIMAT method). The degree of oxidation as a function of time is observed by different methods: concentration of peroxide (AOAC 965.33-Peroxide Value, conjugated diene at 233 nm AOCS Official Method Ti 1a-64), concentration of secondary products such as aldehyde (thiobarbituric acid value AOCS Official Method Cd 19-90), carbonyl (*p*-anisidine value AOCS Official Method Cd 18-90). The direct evaluation of AOC is strongly time consuming and not fully adapted for the evaluation of the AO yield of the extraction from plants or peels of fruits and plants. 

The indirect methods of AO capacity are based on the evaluation of radical exchange capacity and are classified according to their mechanism of action: (1) hydrogen-atom-transfer (HAT)-based assays such as the total radical-trapping antioxidant parameter (TRAP), oxygen radical absorbance capacity (ORAC), and crocin-bleaching assays (CBAs) and (2) electron-transfer (ET)-based assays such as the 2,2-diphenyl-1-picrylhydrazyl assay (DPPH), trolox equivalent antioxidant capacity (TEAC), ferric ion reducing antioxidant power (FRAP), cupric ion reducing antioxidant capacity (CUPRAC), and Folin–Ciocalteu assay [9,10]. These indirect methods are not correlated with each other or with direct evaluation methods of AOC. Other methods have been used for antioxidant detection and quantification, the most common being liquid chromatography (HPLC) coupled to UV or mass spectrometry, and present also several limitations. HPLC-MS devices are relatively expensive, and the related methods are time consuming because they require a tedious sample preparation step. However, most of the above-cited methods are relatively expensive and time consuming, especially when they require sample preparation, and many of them present a lack of specificity [11,12].

To overcome these obstacles, electrochemical methods can be used as an alternative to the conventional techniques. They are simple and fast, involve low-cost and easily accessible instrumentations, and do not require any complicated sample preparation. The electrochemical reduction mimics the electron transfer method (ET), but offers a wider solvent range, even apolar solvents using specific supporting electrolytes, allowing the analysis of non-polar antioxidants, contrary to ET, which uses polar solvents (DPPH-acetone, ethanol, methanol, TEAC and FRAP, CUPRAC in water). Electroanalytical techniques, especially voltammetry (cyclic voltammetry, differential pulse voltammetry), are widely used to evaluate the antioxidant potential in several samples [13,14,15]. Moreover, the use of disposable screen-printed electrodes (SPEs) instead of traditional electrodes implies a decrease of the sample volume used in each experiment and makes the measurements faster and more accurate as the tedious cleaning processes of the electrode are eliminated. With a natural mix of antioxidants, the voltammogram obtained with a plant extract, for example, is complex, without clear peaks of oxidation and reduction. Cyclic voltammetry is adapted to evaluate the content and antioxidant capacity of isolated antioxidant found in wine or to evaluate globally the AOC of a wine [16], but not specifically one antioxidant in a mixture. 

The only issue with the electrochemistry–antioxidant combination is the lack of selectivity (very close oxidation peaks), the instability of the antioxidants, their high reactivity, and the structural similarity between the compounds (for example, caffeic acid, ferulic acid, or hydroxytyrosol). To this end, molecular imprinting is a possible solution, capable of improving the selectivity towards the studied compound. It is a technique that consists of creating synthetic polymers with binding sites complementary to the target molecule, in terms of shape, size, and functional groups. The molecular imprinting technique uses two main strategies: radical polymerization of organic monomers giving a molecular imprinting polymer (MIP) or hydrolysis and condensation of alkoxysilanes by acid or base catalysis giving molecular imprinting silica (MIS). The main reagents to synthetize MIPs are a template, which is most often the target molecule, a functional monomer, a cross-linker, an initiator, and a solvent. Their choices are based on the polymerization process used for the polymer synthesis. The majority of the imprinted polymers are acrylate-based molecularly imprinted polymers (MIPs), synthesized by radical polymerization, commonly using methacrylic acid (MAA) as a functional monomer. On the other hand, molecularly imprinted silicas (MISs) are synthesized by the sol–gel process, based on two steps: hydrolysis and condensation. The main functional monomers are alkoxysilane molecules, such as (3-Aminopropyl)-triethoxysilane (APTES) and (3-Aminopropyl)-trimethoxysilane (PTMOS) [6,17].

Molecularly imprinted polymers were initially used as solid phase extraction materials (MISPEs) to replace immunosorbents [18,19] and aptamers because of their inexpensive and simple preparation, as well as for their high thermal and chemical stability [20,21]. They have been used for the analysis of several compounds, such as drugs [22,23], pesticides [24,25], industrial contaminants [23,24,25,26], etc., mostly coupled with high-performance liquid chromatography (HPLC). Then, the application of MIPs and their designs have evolved, and they have been used for antioxidant detection in many sectors (drugs, foods, biological fluids, etc.) [6]. The combination of MIPs with electrochemical techniques, especially voltammetry, has been applied for antioxidant detection, such as caffeic acid in wine, using different types of electrodes. For example, Reference [27] modified a platinum electrode with (poly(3,4-ethylenedioxy) thiophene); Reference [28] modified a screen-printed carbon electrode with graphene oxide by electrochemical reduction; Reference [29] used a nitrogen-doped carbon-modified glassy carbon electrode for the determination of caffeic acid in wine samples. The voltammetry analysis of one antioxidant in wine is challenging due to the numerous antioxidants in wine, 365 molecules with AOC identified in white wine [30], maybe much more in red wine, for instance hydroxybenzoic acids such as caffeic acid, stilbenes such as resveratrol, and polyphenols such as flavonols, flavonoids, anthocyanidins, anthocyanins, or tannins [31,32].

The main objective of this work was to combine cyclic voltammetry and MIPs, in order to implement a new method for the specific detection of caffeic acid in a hydroalcoholic medium such as wine. This new method should be simpler and easier than those previously developed. It should not imply any interference with other antioxidants. For this reason, an MIP specific to CA was synthesized then characterized by FTIR, laser diffraction granulometry, nitrogen adsorption, and scanning electron microscopy. Several parameters were studied during the development of this new CA-MIP/CV method: pH, polymer mass, and selectivity. The final step was the utilization and the validation of this new method for a Burgundy red wine.

## 2. Materials and Methods

### 2.1. Chemicals

Caffeic acid (≥98%, CAS number 331-39-5), *N*-phenylacrylamide (*N*-PAA 99%, CAS number 2210-24-4), ethylene glycol dimethacrylate (EGDMA 98%, CAS number 97-90-5), azobisisobutyronitrile (AIBN 98%, CAS number 78-67-1), *p*-coumaric acid (≥98%, CAS number 501-98-4), gallic acid (97.5–102.5 titration, CAS number 149-91-7), trans-ferulic acid (≥99%, CAS number 537-98-4), sinapic acid (≥98%, powder, 530-59-6), acetonitrile (ACN, gradient grade, ≥99%, CAS number 75-05-8), methanol (gradient grade, ≥99%, CAS number 67-56-1), phosphate-buffered saline (PBS tablets), ethanol (EtOH, absolute, CAS number 64-17-5), and acetic acid (glacial, CAS number 64-19-7) were purchased from Sigma Aldrich, France.

The water used in all experiments was deionized and obtained from an Elga Ionic system PURELAB Option.

### 2.2. Instrumentation

All the voltammetric measurements were carried out using a portable and easy-to-handle μStat-i 400 potentiostat, connected to a PC, and screen-printed carbon electrodes, all purchased from Metrohm, France. The analyses were performed with a scan rate of 50 mv/s, between −0.4 and +0.8 V. The measured intensity (anodic peak intensity, I_pa_) is proportional to the concentration of the detected compound in the medium. 

For the morphological characterization, Fourier transform infrared (FTIR) spectroscopy was performed on a PerkinElmer spectrum 65 FT-IR spectrometer in the range 4000–500 cm^−1^ using attenuated total reflectance sampling. Sixty-four scans with a resolution of 4 cm^−1^ were applied.

The Brunauer–Emmett–Teller (BET) method was used for surface area analysis and was performed by nitrogen adsorption–desorption at a temperature of 77 K using an ASAP 2020 instrument. Prior to the analysis, 50 mg of the sample was outgassed for 14 h at room temperature.

Scanning electron microscopy (SEM) was applied for the surface morphological characterization using a Zeiss Gemini SEM 500 apparatus (Oberkochen, Germany). A SE2 Everhart-Thornley was used as the detector.

### 2.3. Synthesis of the Molecularly Imprinted and Non-Imprinted Polymers

The molecularly imprinted polymer specific for caffeic acid (CA MIP) was prepared at room temperature and then synthesized under thermal conditions. The template molecule, caffeic acid (CA, 0.5 mmol), was solubilized in methanol, then acetonitrile was added (50 mL, 20/80 *v*/*v*, respectively). While stirring, the functional monomer (*N*-PAA, 2 mmol) was added, followed by the cross-linker (EGDMA, 10 mmol) and then the initiator (AIBN, 0.44 mmol). The mixture was degassed by nitrogen sparging. In parallel, non-imprinted polymer (NIP) was prepared under the same conditions as CA MIP, but without adding the template. The reaction mixtures were placed in an incubator under 60 °C for 24 h. Afterwards, the polymer was separated from the solution by centrifugation at 10,000× *g* for 10 min at 20 °C and then washed with a 10% (*m*/*v*) acetic acid solution with ethanol in order to eliminate the template from the polymer. Several washing steps were required until the caffeic acid was no longer detectable by HPLC. After washing, the polymers were dried for 6 h at 60 °C. They were stored at room temperature until use.

### 2.4. Effect of pH on Electrochemical Detection of CA in Hydroalcoholic Medium 

Two calibration curves were constructed over the range of 0.06 mM to 2.78 mM of caffeic acid. Six standards were prepared in an aqueous ethanolic buffer, aqueous-phosphate-buffered (PBS 0.05 M)/ethanol (EtOH 90/10, *v*/*v*) solution, at pH 3 and pH 7, and voltammetric measurements were applied. All samples were prepared in triplicate.

### 2.5. Rebinding Experiments of CA Using MIP and NIP in Hydroalcoholic Medium: Effect of pH and Polymer Mass

Rebinding properties (steps represented in Figure S1 of the Supplementary Material) were studied by adding the CA MIP to 1 mL of PBS 0.05 M/EtOH (90/10, *v*/*v*) solution, containing 1.11 mM of CA, varying the pH (pH 3 and pH 7) or varying the mass of the CA MIP, ranging from 10 mg to 50 mg. Eppendorf tubes were agitated for 2 h at room temperature using a Stuart rotator SB2. The same procedure was performed with the NIP, all in triplicate. After separating the polymer from the liquid by centrifugation at 10,000× *g* for 10 min at room temperature, the supernatant was deposited on the surface of a screen-printed carbon electrode attached to the potentiostat, and CV analyses were carried out. The obtained voltammograms were directly related to the amount of CA remaining in the solution and that was not sorbed by the CA-MIP.

The concentration of CA retained by the CA-MIP (C_ads_, mM) was calculated by the difference between the initial amount of CA concentration (C_0_, mM), initially added to the solution, and the remaining CA concentration (C, mM) in the supernatant. The amount of retained or adsorbed CA (N_ads_, µmol) was calculated based on the concentration (C_ads_).

The time of contact between the polymers and the solution was determined based on a kinetic study in which nine samples were prepared in the same previous solution containing 1.11 mM of caffeic acid and 20 mg of polymer, CA MIP or NIP. The supernatants were evaluated at various times ranging from 1 min to 4 h. The rebinding was so fast that even at one minute, the amount of retained caffeic acid reached about 0.78 mM, and by 4 h, it was 0.83 mM (Figure S2). The selected contact time was 2 h of stirring (0.80 mM), as it represents the time where the kinetics became stable.

### 2.6. Selectivity Studies

Ten milligrams of CA MIP or NIP was contacted in Eppendorf tubes (in triplicate), with 1 mL of PBS 0.05 M/EtOH (90/10, *v*/*v*, pH 3) solution containing 0.55 mM of caffeic acid, sinapic acid, *p*-coumaric acid, ferulic acid, or gallic acid, separately. The tubes were then agitated for 2 h and finally centrifuged for 10 min at 10,640× *g* at room temperature. The supernatants were analyzed by cyclic voltammetry using the same method described in Section 2.5 in order to determine the amount of caffeic acid or its interferents retained by the polymers. 

A calibration curve was constructed over the range of 0.06 to 0.55 mM of each phenolic acid.

### 2.7. Preparation and Electrochemical Analysis of CA in Wine Samples

A sample of Burgundy red wine (Pinot Noir, 2018) was purchased at a local supermarket. The proposed method was tested to determine the CA in the wine using the same previously described procedure (Section 2.5). The wine was diluted 10 times in PBS 0.05 M/EtOH (90/10, *v*/*v*, pH 3), and 10 mg of CA-MIP or NIP was added to 1 mL of the diluted wine. Analytical recovery experiments were also performed after spiking four different samples with 0.17, 0.22, 0.28, and 0.42 mM of caffeic acid. All samples were prepared in triplicate. Voltammograms were extracted in order to determine the rebinding properties and the efficiency of the method.

A calibration curve was constructed over the range 0.06 mM to 1.67 mM of caffeic acid in the diluted wine. The calibration curve equations and the correlation coefficients were y = 35.3x + 1.80 and *R*^2^ = 0.995 (y is I_pa_ in µA, and x is caffeic acid concentration in mM).

### 2.8. HPLC Determination of CA in Wine Samples

The CA in the wine samples was analyzed by an alternative HPLC method using a C18-column on a Shimadzu LC equipped with a UV–visible absorbance detector (Kyoto, Japan). The determination was performed using a gradient of the mobile phase: acetonitrile/water. The flow rate was 1.0 mL.min^−1^, and the detection was performed at 325 nm. The same samples prepared for the electrochemical analysis in Section 2.7 were also prepared for HPLC, then filtrated and injected. 

A calibration curve was constructed over the range 0.06 mM to 1.67 mM of caffeic acid in the diluted wine. The calibration curve equations and the correlation coefficients were y = 114432.x + 60205 and *R*^2^ = 0.9964 (y is I_pa_ in µA, and x is caffeic acid concentration in mM).

## 3. Results and Discussion

### 3.1. Synthesis of the Molecularly Imprinted CA-MIP and Non-Imprinted Polymer 

**Figure 1:**
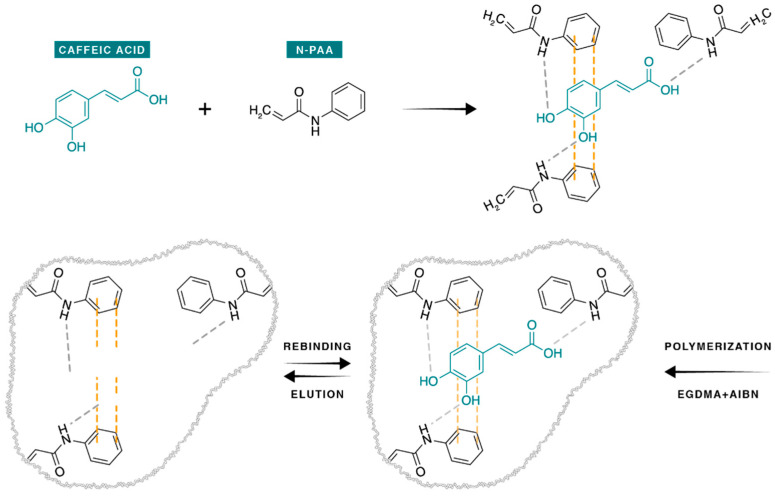
Proposed chemical reactions and interactions involved in the CA-MIP synthesis.

The synthesis of the polymers was based on radical polymerization, in which the functional monomer binds to the CA through its functional groups (dipole–dipole interactions and π-π stacking) and to the cross-linker in order to constitute the polymer (Figure 1). The polymerization was initiated by the AIBN under the thermal effect. The ratio of the template to functional monomer and cross-linker used was 1:4:20 based on previous MIP synthesis performed by our group [33]. For the solvent choice, several studies used a mixture of acetonitrile and toluene [34] for the synthesis of MIP specific to CA. Toluene allows the solubilization of caffeic acid, but given its high toxicity, it was replaced by methanol in this study.

### 3.2. Morphological Characterization

The CA MIP and NIP were characterized by Fourier transform infrared (FTIR) spectroscopy, scanning electron microscopy, and the BET method. Results and figures are presented in the Supplementary Materials. 

The FTIR spectrum (Figure S3) of both polymers showed that all the observed peaks were related to the absorption of the EGDMA cross-linker and the *N*-phenylacrylamide monomer after the radical polymerization. The main absorption bands were at: 1740 cm^−1^ (C=O group in EGDMA and N PAA), 1461 cm^−1^ and 1409 cm^−1^ (aromatic ring in *N*-PAA), and 1289 cm^−1^ and 1180 cm^−1^ (C-O-C group in EGDMA).

BET was applied to determine the specific surface area of the sample. For CA-MIP, it was 9.9 m^2^.g^−1^, and for NIP, it was 8.3 m^2^.g^−1^.

SEM micrographs (Figure S4) showed a bigger particle size for CA-MIP than NIP, which ranged from 381 to 427 nm.

### 3.3. Effect of pH on CA Detection by Voltammetry

**Figure 2:**
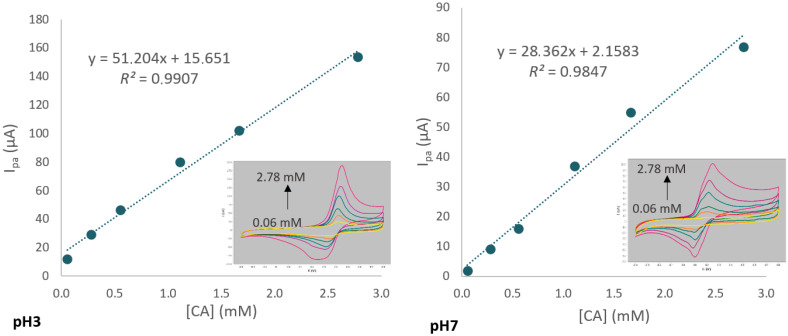
Calibration curves for caffeic acid (CA) in PBS 0.05 M/EtOH (90/10, *v*/*v*) solution, at pH 3 (left) and pH 7 (right). Inset: CV for caffeic acid at different concentrations, ranging from 0.06 to 2.78 mM of CA. The critical value of Pearson’s correlation coefficient is 0.974 for 6 points (degrees of freedom: 4) and a level of significance for a two-tailed test of 0.1%.

The choice of pH is a major factor in caffeic acid detection. Figure 2 represents the calibration curves and their corresponding cyclic voltammograms (inset) and shows that at pH 3, the intensity was greatly increased compared to pH 7. For example, at 2.78 mM of caffeic acid, the intensity was 153.5 µA at pH 3 and 76.8 µA at pH 7.

As explained by Genaro-Mattos et al. [35], this could be related to the structure of caffeic acid, which has three ionizable hydroxyl groups: carboxyl group (pKa = 4.8), *p*-hydroxy group (pKa = 8.6), and m-hydroxy group (pKa = 11.2) (Figure S5). The increase of the pH in the medium results in the deprotonation of caffeic acid, which decreases its interaction with the functional monomer. At pH 7, where the pH is higher than the pKa of the carboxylic group, the carboxylic ion is the major species present in the solution. The deprotonated species are less electroactive than the protonated ones.

### 3.4. Effect of pH on the Rebinding Properties of CA

Testing the effect of pH on caffeic acid by cyclic voltammetry in the presence of CA-MIP is important; it helps to understand if there is any matrix effect exerted by the polymer. The same measurements performed with cyclic voltammetry as Figure 2 were conducted in the presence of caffeic acid (CA), but this time in addition to the imprinting polymer, CA-MIP, and non-imprinting polymer, NIP. The difference in intensity at the anodic peak (I_pa_) between CA alone and CA with CA-MIP or NIP allows the calculation of the absorption of CA by the CA-MIP and NIP.

**Figure 3:**
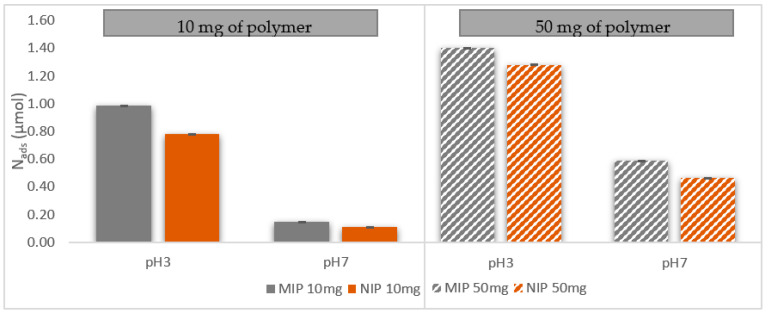
pH effect on the quantity of caffeic acid adsorbed (N_ads_) by CA MIP using cyclic voltammetry in a PBS 0.05 M/EtOH (90/10, *v*/*v*) solution containing 1.11 mM of CA, in the presence 10 mg (left) and 50 mg (right) of CA-MIPCA and NIP.

At pH 3, the intensity of the signal is greatly increased, and so is the reuptake activity of the polymer. As shown in Figure 3, the quantity of caffeic acid adsorbed at pH 3, in the presence of 10 mg or 50 mg of polymer, is more important than the quantity adsorbed at pH 7. This confirms the explanation proposed in Section 3.3 and that the structure of caffeic acid could be the reason for these differences. 

### 3.5. Effect of the Polymer Mass on the Rebinding of CA

**Figure 4:**
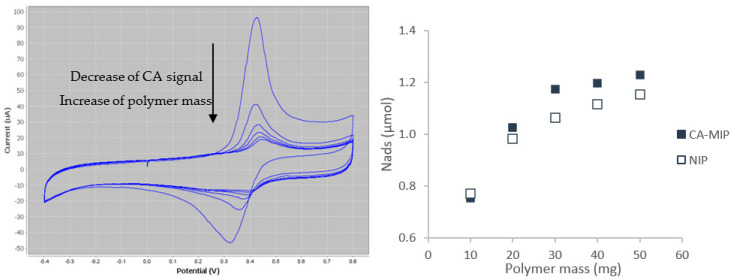
Left: CV for caffeic acid (CA) remaining in the medium after adding different quantities of CA-MIP (0, 10, 20, 30, 40, and 50 mg). Scan rate: 50 mV/s; the arrow shows the reduction of I_pa_ and the remaining caffeic acid with increasing the content of imprinting polymer CA-MIP. Right: effect of the polymer mass on the amount of adsorbed caffeic acid in a PBS 0.05 M/EtOH (90/10, *v*/*v*) solution containing 1.11 mM of caffeic acid. The error bar is 0.15% for all samples.

Figure 4 shows (a) the CV responses of the SPCE, after dropping on its surface 50 µL of the supernatant containing caffeic acid, with progressive addition of CA-MIP, at pH 3, and (b) the amount of caffeic acid retained by the polymer CA-MIP.

The decrease of the anodic peak current, I_pa_ (Figure 4), is a result of the increase of the added polymer mass. As shown in Figure S2, the reuptake activity was relatively fast. At 10 mg, 62.6% of the added caffeic acid was already adsorbed. A slight difference can be observed between the imprinting CA-MIP and non-imprinting NIP responses. This could be explained by the fact that the MIP has chemical and steric complementarity with the target molecule (CA). However, the NIP has only chemical complementarity with CA. The chemical complementarity is due to the functional monomer, which is present in both the MIP and NIP. Steric interactions result from the template molecule, which, after washing, leaves specific cavities in the polymers.

### 3.6. Selectivity Studies

**Figure 5:**
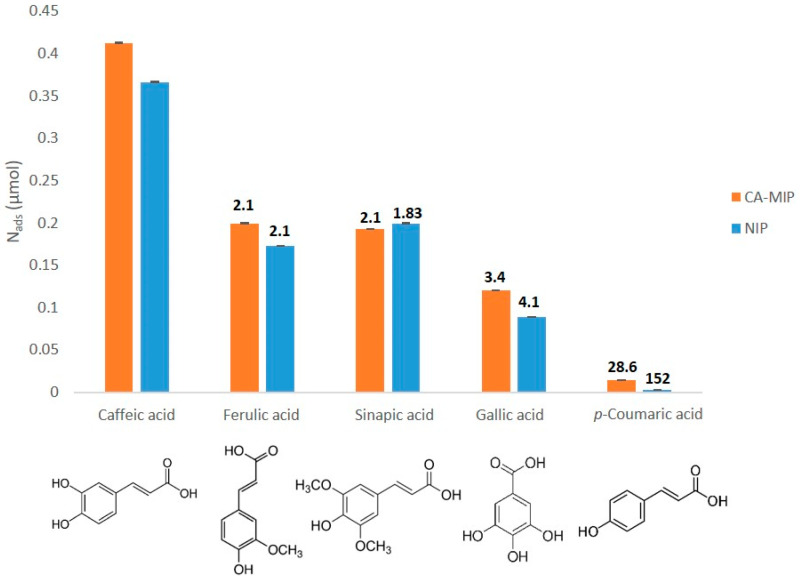
Relative amount of caffeic acid and its interferents adsorbed by the CA-MIP and NIP in a PBS 0.05M/EtOH (90/10, *v*/*v*) solution containing 0.55 mM of each compound, in the presence of 10 mg of MIP or NIP. The selectivity coefficient α is represented in bold above every bar. Interferents: ferulic acid, sinapic acid, gallic acid, and *p*-coumaric acid.

In order to study the selectivity of the CA-MIP, cyclic voltammograms were obtained after dropping 50 µL of each solution, containing different antioxidants with a very close structure to caffeic acid, named interferents. The selectivity coefficient α was calculated for each interferent as follows:

(1) $$\alpha =\frac{N_{ads}CA}{N_{ads}\;interferent}$$

where *N_ads_ CA* is the amount of adsorbed caffeic acid and *N_ads_* interferent is the amount of adsorbed interferent by the polymer. As shown in Figure 5, the CA MIP had the highest selectivity for caffeic acid: 2.1-times greater than for ferulic acid and sinapic acid, 3.4-times than for gallic acid, and 28.6-times than for *p*-coumaric acid. This high selectivity can be explained by the arrangement of the functional groups matched with *CA* inside the polymer. The ortho-diphenol structure of *CA* could be behind this high selectivity.

### 3.7. Application to Wine, Validation, Recovery Tests, and Limit of Detection

The binding properties were studied for a Burgundy red wine, where the CA MIP was applied for CA determination using the new cyclic voltammetry (*CV*) method. The analytical recovery was calculated using the following equation, and the results are presented in Table 1:

(2) $$Recovery=\frac{{\left[CA\right]}_{found}}{{\left[CA\right]}_{added}}\times 100$$

The obtained percentages for the *CV* method ranged between 79 and 89%. These values imply that the matrix has little influence on the CA-MIP activity and are acceptable for trace analysis in a real sample matrix. A limit of detection (LOD) of 0.06 mM and a limit of quantification (LOQ) of 0.11 mM were respectively determined for the CV-MIP-CA method using the following formulas:

(3) $$LOD=\frac{\left(intercept+3s\right)}{slope}$$

(4) $$LOQ=\frac{\left(intercept+10\;s\right)}{slope}$$

where the intercept and the slope were obtained from the calibration curve determined for the wine and “s” corresponds to the standard deviation of the intercept (Figure S6).

To validate the newly developed cyclic voltammetry method using CA-MIP, an alternative HPLC-UV method was used to analyze the same wine samples. The concentrations of CA found in these same wine samples by HPLC-UV are presented in Table 1. The recovery percentages determined by HPLC were very close to those determined by the MIP-CA/CV method. A good correlation between the CA concentration measured by CA-MIP/CV and the CA concentration measured by HPLC-UV was obtained (*R*^2^ = 0.9973) (Figure S7). The recovery values obtained with each technique ranged between 79 and 89% (CV) and between 81 and 89% (HPLC-UV). The fact that the values were lower than 100% could be explained by the instability of the antioxidants, which are usually influenced by light and temperature, thus the loss of the compound during the experiment. 

## 4. Conclusions

A highly selective CA-MIP was developed and combined with cyclic voltammetry and was successfully applied to the determination of caffeic acid in wine samples. This indirect method is fast and only requires simple and portable instrumentation. The kinetics tests showed that rebinding happened in the very first minutes. Under the optimized analytical conditions, the signal was more important at pH 3 due to the structure of CA. The selectivity of the MIP toward CA compared to other similar compounds was due to the interactions between the template and the functional groups of the functional monomer (hydrogen bonds and π–π stacking). In wine samples and under the optimized analytical conditions, the anodic peak current was linear to the CA concentration from 0.17 to 0.56 mM with an LOD of 0.06 mM and an LOQ of 0.11 mM. The CV method was validated by a conventional method, HPLC-UV, showing good correlation between the recoveries (*R*^2^ = 0.9973). Consequently, this indirect method applied with CA-MIP is a fast, simple, accurate, and selective method for CA determination in wine samples. This method could facilitate the detection of polyphenols in food matrices and, thus, could be used in several industrial applications.

## Figures and Tables

**Table 1:** Added and recovered concentration of CA from wine samples using the MIP-CA/cyclic voltammetry and the HPLC-UV methods.

		Cyclic Voltammetry		HPLC-UV	
Wine Sample	Added (CA) (mmol/L)	Found (CA) (mmol/L)	Recovery (%)	Found (CA) (mmol/L)	Recovery (%)
No CA added	0	0.009 ± 0.004	-	0.010 ± 0.002	-
1st level	0.167	0.149 ± 0.009	89	0.141 ± 0.005	88
2nd level	0.222	0.187 ± 0.01	84	0.183 ± 0.008	89
3rd level	0.278	0.221 ± 0.008	79	0.230 ± 0.019	83
4th level	0.416	0.337 ± 0.014	81	0.343 ± 0.013	81

## Data Availability

The data presented in this study are available in the article and Supplementary Materials.

## Supplementary Materials

The following Supporting Information can be downloaded at: https://www.mdpi.com/article/10.3390/antiox11102036/s1. Figure S1: Preparation steps of rebinding experiments using CA-MIP/NIP and measured by CV; Figure S2: Kinetic study representing the amount of adsorbed caffeic acid from 1 min to 4 h of stirring, applied to nine samples of 1 mL containing 1.11 mM of caffeic acid in the presence of 20 mg CA-MIP (error bars are smaller than the symbol); Figure S3: ATR-FTIR spectra for CA-MIP and NIP; Figure S4: SEM images of CA-MIP and NIP at different scales; Figure S5: Deprotonation of caffeic acid as a function of increasing pH and the respective pKa values; Figure S6: Calibration curve for the determination of CA in red wine using the MIP-CA/CV method. The wine was diluted 10 times in a PBS 0.05 M/EtOH (90/10, *v*/*v*, pH 3), and 10 mg of CA-MIP was added to 1 mL of the diluted wine. Cyclic voltammetry parameters: voltage range of −0.4 to 0.8 V at a scan rate of 50 mV/s versus Ag/AgCl; Figure S7: Correlation between the MIP-CA/Cyclic voltammetry method and the HPLC-UV method for the determination of CA in wine.
