# Supplementary material for: Combination of Screen-Printed Carbon Electrode and Molecularly Imprinted Polymers for the Selective Determination of Phenolic Compounds in Wine
Source: Antioxidants (Basel). 2022 Oct 15;11(10):2036. doi: 10.3390/antiox11102036 (PMC9598643; doi:10.3390/antiox11102036)
Supplement: Supplementary file 1 [file antioxidants-11-02036-s001.zip › antioxidants-1919473-supplementary.pdf]

SUPPLEMENTARY MATERIAL

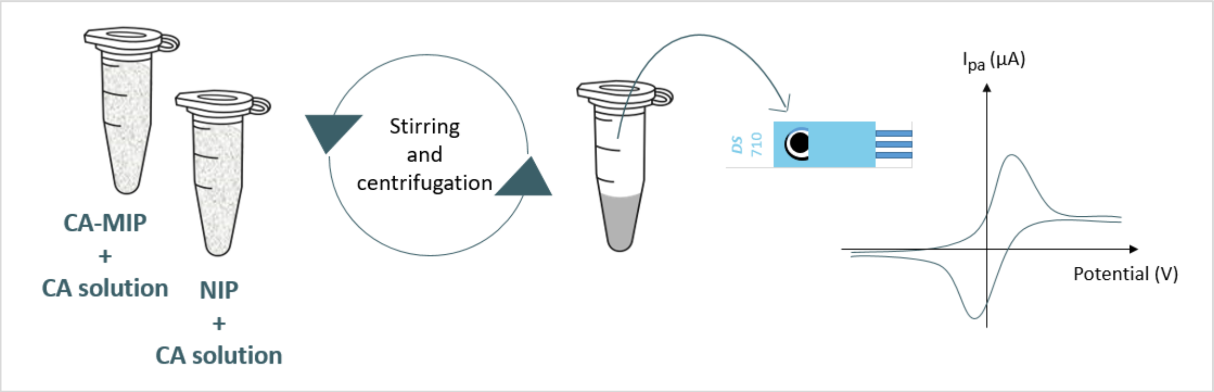

Figure S1. Preparation steps of rebinding experiments using CA-MIP/NIP and measured by CV.

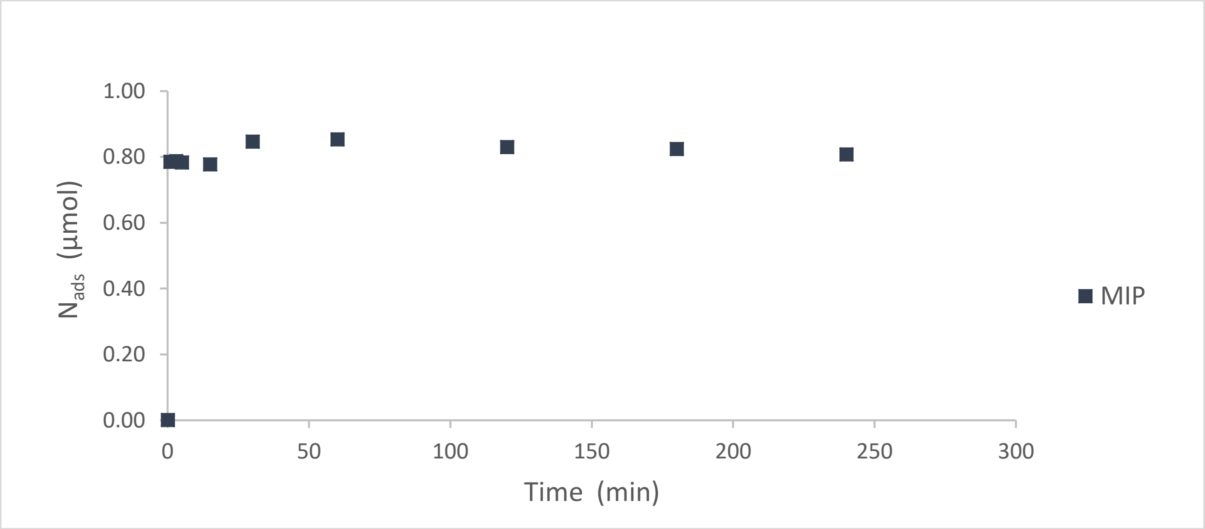

Figure S2. Kinetic study representing the amount of adsorbed caffeic acid from 1 min to 4 h of stirring, applied with nine samples of 1 mL containing 1.11 mM of caffeic acid in the presence of 20 mg CA-MIP (error bars are smaller than the symbol).

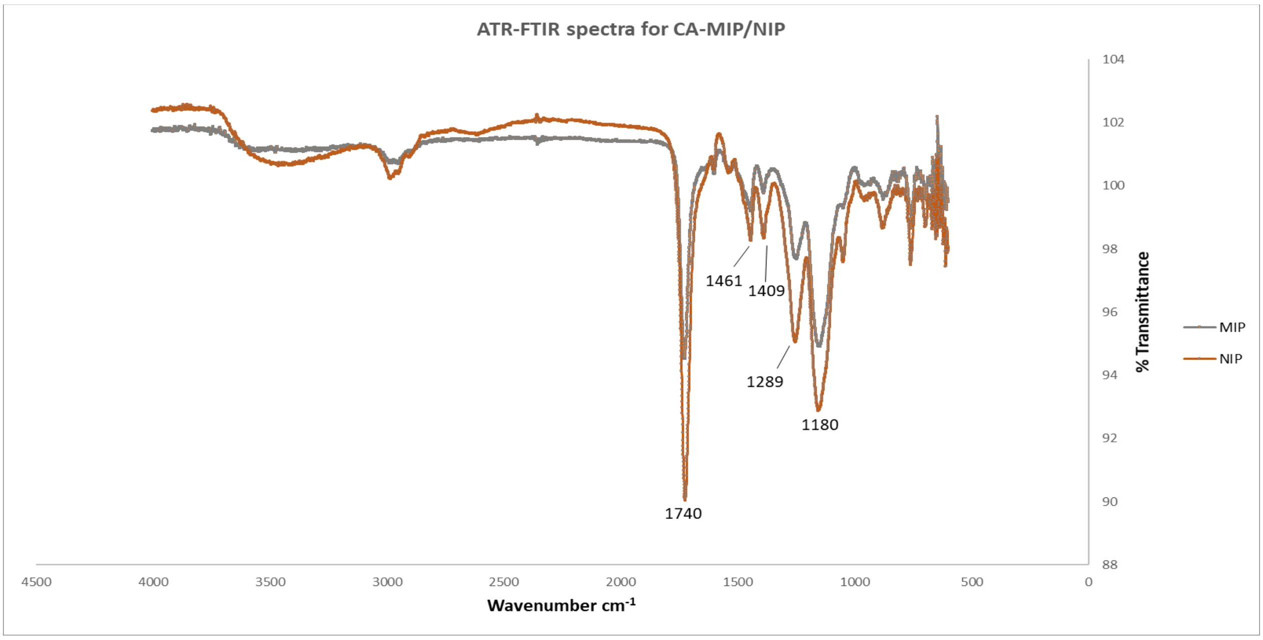

Figure S2. ATR-FTIR spectra for CA-MIP and NIP

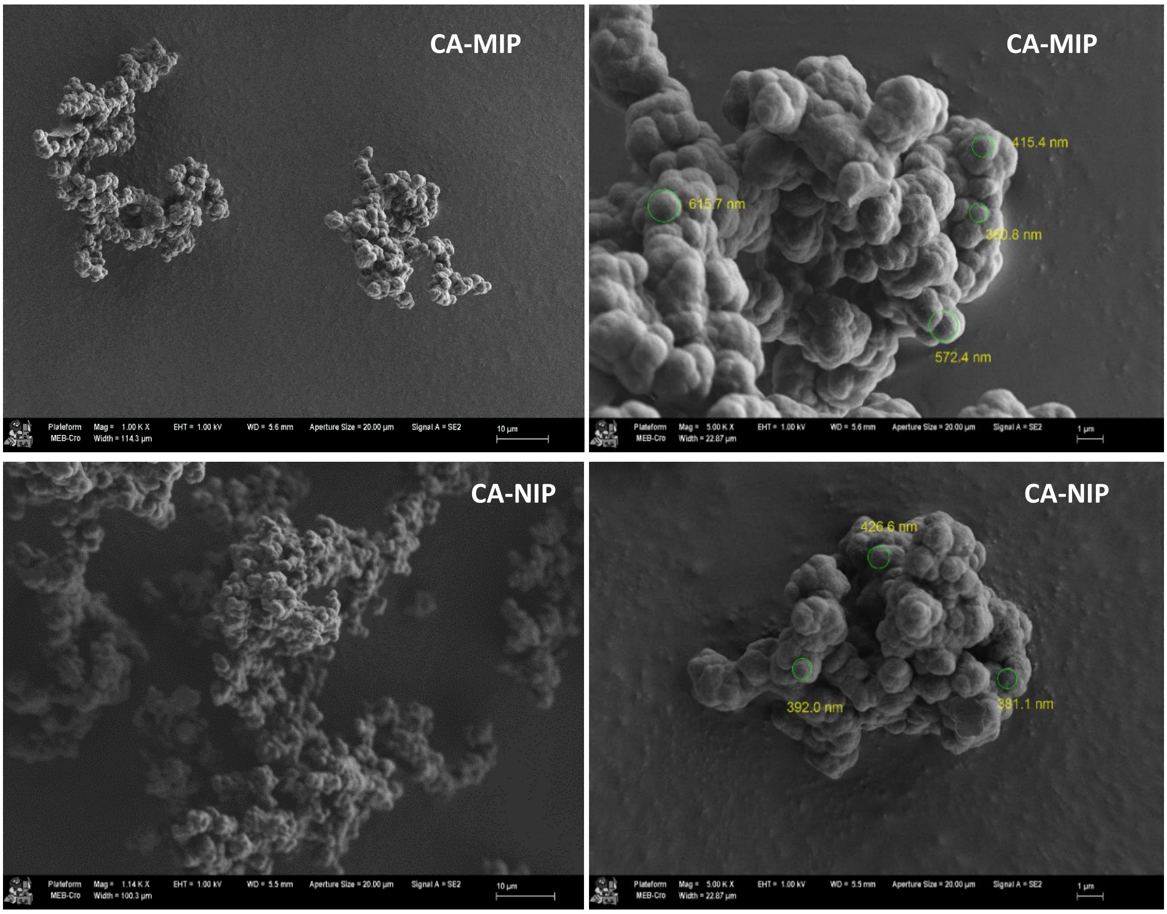

Figure S3. SEM images of CA-MIP and NIP at different scales

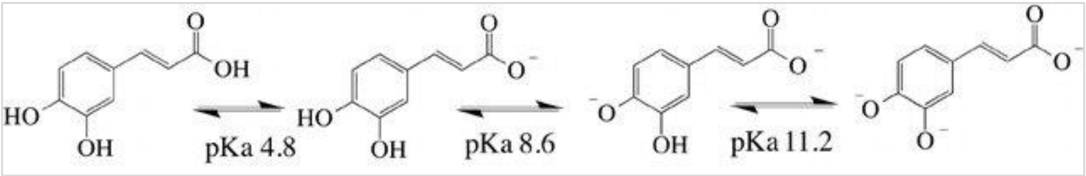

Figure S4. Deprotonation of caffeic acid as a function of increasing pH and the respective pKa values. [35]

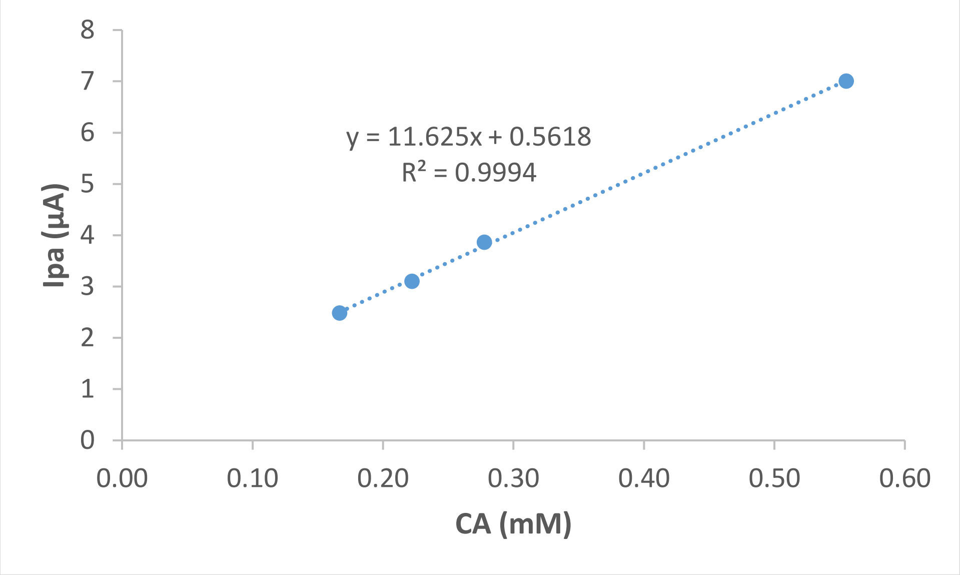

Figure S5. Calibration curve for the determination of CA in red wine using the MIP-CA/CV method. The wine was diluted 10 times in a (PBS 0.05 M/ EtOH) (90/10, *v/v*, pH 3), and 10 mg of CA-MIP were added to 1 mL of the diluted wine. Cyclic voltammetry parameters: voltage range of −0.4 to 0.8 V at a scan rate of 50 mV/s versus Ag/AgCl.

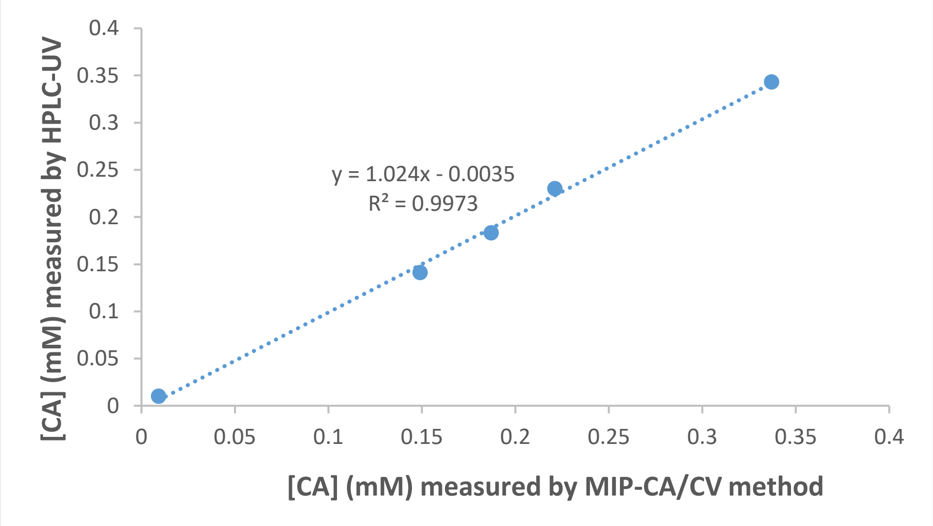

Figure S6.Correlation between MIP-CA/Cyclic voltammetry method and HPLC-UV method for the determination of CA in wine.
